# Supplementary material for: A review on the traditional uses, nutritive importance, pharmacognostic features, phytochemicals, and pharmacology of Momordica cymbalaria Hook F
Source: PeerJ. 2024 Feb 27;12:e16928. doi: 10.7717/peerj.16928 (PMC10906271; doi:10.7717/peerj.16928)
Supplement: Supplemental Information 1 [file peerj-12-16928-s001.pdf]

A Review to Focus on The Traditional Uses, Nutritive Importance, Pharmacognostical Features, Phytochemicals and Pharmacology of *Momordica cymbalaria* Hook F.

Sayeed Mohammed Firdous[1*], Dinesh Babu[2], Zainab Irfan[3], Marwa A.A.Fayed[4*]

[1*] Department of Pharmacology, Calcutta Institute of Pharmaceutical Technology and Allied Health Sciences, Uluberia, Howrah-711316, West Bengal, India.

[2] Faculty of Pharmacy and Pharmaceutical Sciences, Katz Group Centre for Pharmacy and Health Research, University of Alberta, Edmonton, Alberta, T6G 2R3, Canada.

[3] Department of Pharmaceutical Technology, Brainware University, Kolkata-700125, West Bengal, India.

[4*] Department of Pharmacognosy, Faculty of Pharmacy, University of Sadat City, Sadat City 32897, Egypt.

[*]**Corresponding Authors**:

- Sayeed Mohammed Firdous

Associate Professor, Department of Pharmacology, Calcutta Institute of Pharmaceutical Technology and Allied Health Sciences, Uluberia, Howrah-711316, West Bengal, India

E-mail: firdous.oncology@gmail.com, https://orcid.org/0000-0002-7538-987X

- Marwa A.A.Fayed

Department of Pharmacognosy, Faculty of Pharmacy, University of Sadat City, Sadat City 32897, Egypt, marwa.fayed@fop.usc.edu.eg, https://orcid.org/0000-0001-5609-7436

Table S1: Nutrients composition of *Momordica cymbalaria* and *Momordica charantia*

Constituents (mg/100 gm)	*Momordica cymbalaria*	*Momordica charantia*
Moisture	84.30	83.20
Fibers	6.42	1.70
Carbohydrate	12.60	10.60
Protein	2.15	2.10
Calcium	372.00	23.00
Potassium	500.00	171.00
Sodium	40.00	2.40
Iron	1.70	2.00
Copper	0.18	0.19
Manganese	0.32	0.08
Zinc	0.82	0.46
Phosphorus	0.46	38.00
Vitamin C	290.00	96.00
β-Carotene	0.01	126.00

Table S2: Ash values of tubers of *Momordica cymbalaria*

Parameters	Values % (w/w) ± SD
Total ash	3.86 ± 0.004
Acid-insoluble ash	1.26 ± 0.01
Water-soluble ash	2.90 ± 0.002
Sulfated ash	1.26 ± 0.014

Table S3: Fundamental phytochemical screening of tubers of *Momordica cymbalaria* (C. Gopu & Taduri, 2021).

Phytochemicals	Petroleum ether	Chloroform	Ethanol	Water
Alkaloids	+	--	+	--
Sterols	+	+	--	--
Triterpenes	+	+	+	--
Saponins	--	--	+	+
Flavonoids	--	--	+	--
Tannins	--	--	--	--
Fixed oils and fats	--	--	--	--
Gum and mucilage	--	--	--	--

Carbohydrate	--	--	+	+
Cardiac glycosides	--	--	+	+
Proteins and amino acids	--	--	--	--

+ = presence and --= absence of photochemical.
